# Rational Design of Single-Atom Catalysts for the Hydrogen Evolution Reaction for Advancing Sustainable Energy Conversion and Storage

**DOI:** 10.3390/nano16211448

**Published:** 2026-11-08

**Authors:** Sandip Maiti, Dongwoo Shin, Sumanta Sahoo, Jaehyun Hur

**Affiliations:** 1 School of Chemical, Biological, and Battery Engineering, Gachon University, Seongnam-daero, Sujeong-gu, Seongnam-si 13120, Gyeonggi-do, Republic of Korea; 2 Department of Chemical Engineering, Yeungnam University, 280 Daehak-ro, Gyeongsan-si 38541, Gyeongsangbuk-do, Republic of Korea

**Keywords:** hydrogen evolution reaction, single-atom catalysts, morphological investigations, structural relationships, catalytic activity and stability, DFT studies

## Abstract

Progress in sustainable hydrogen production requires electrocatalysts that accelerate the hydrogen evolution reaction (HER) with excellent catalytic activity and durability. Single-atom catalysts (SACs) have gained attention as superior HER electrocatalysts because of their almost 100% atomic utilization, distinct coordination surroundings, and tunable electronic structures. This review surveys advances in HER SACs with emphasis on atomic-level engineering and catalytic mechanisms. We first explore the fundamental principles and reaction mechanisms of the HER. The morphological engineering of numerous SAC architectures is discussed, along with their structural features and roles in boosting HER performance. The structural characteristics of SACs are examined to explain the roles of orbital interactions, charge redistribution, local structural asymmetry, and metal–support interactions in regulating hydrogen adsorption and HER kinetics. Finally, the catalytic performance of several SAC structures is discussed extensively, with a focus on HER activity, reaction kinetics, and prolonged stability. Density functional theory (DFT) is used to elucidate the mechanism underlying the outstanding catalytic performance of SACs in the HER. Moreover, key challenges associated with scalability, catalyst stability, and long-term operation at practical current densities are discussed, and future research directions are proposed. Finally, we discuss the rational development of next-generation SACs toward sustainable hydrogen production.

## 1. Introduction

The expansion of sustainable and low-carbon energy technologies has become urgent owing to universal energy demand and growing environmental concerns [1,2]. Owing to the limitations of fossil-fuel-based energy sources, such as their extensive greenhouse gas release and ecological pollution, the evolution toward renewable energy sources is gaining increasing attention [3,4]. Hydrogen is considered a promising clean energy carrier owing to its carbon-free utilization, excellent gravimetric energy density, and potential for integration with renewable electricity [5,6]. In particular, hydrogen is produced using renewable electricity, and water (H_2_O) can deliver a sustainable path toward low-carbon energy conversion and storage. Therefore, there is a need to develop highly efficient electrocatalytic systems for renewable hydrogen production and sustainable energy technologies [7,8].

Electrochemical water splitting has received substantial attention among several hydrogen production technologies because it can directly convert renewable electrical energy into the chemical energy stored in hydrogen [9,10]. Water electrolysis has two corresponding half-reactions: the HER at the cathode and the oxygen evolution reaction (OER) at the anode [11,12]. Compared with the OER, the HER usually undergoes faster reaction kinetics. However, their effectiveness strongly depends on the nature of the electrocatalyst and the reaction environment. Sluggish reaction kinetics are strongly related to water dissociation, hydrogen adsorption, and hydrogen formation [13,14]. Nevertheless, this can result in significant overpotential and energy consumption, particularly in alkaline media. Therefore, the development of highly active, ultra-stable and low-cost HER electrocatalysts is essential to reducing the energy requirements of water electrolysis and allowing for practical hydrogen production.

Over recent decades, several studies have been conducted to develop efficient nanostructured electrocatalysts for the HER [15,16,17,18]. Pt-based electrocatalysts exhibit excellent intrinsic HER activity owing to their promising hydrogen adsorption energetics [19,20]. However, the high cost and low abundance of noble metals hinder their large-scale application. In addition, transition-metal-based nanoparticles and heterostructured materials have been widely reported to decrease noble-metal utilization and improve HER catalytic performance [21,22,23,24]. The nanoscale engineering of electrocatalysts enhances the electrochemically available surface area, exposes numerous active sites, and facilitates charge and mass transport. Additionally, the nanoparticle composition, morphology, surface defects, crystal structure, and heterointerfaces can be engineered to control the adsorption energies of key HER intermediates and boost catalytic kinetics.

Despite these advantages, nanoparticle-based electrocatalysts have numerous intrinsic restrictions [25,26,27]. A significant fraction of metal atoms is positioned within the inner side and are therefore unreachable by the reactants, resulting in inefficient utilization of the active metal species. Nanoparticles can undergo accumulation, dissolution, surface reconstruction, or phase transformation during long-term electrochemical operation, resulting in activity degradation and reduced durability. Furthermore, noble-metal nanoparticles often require comparatively high metal loadings, which increase catalyst cost and restrict their practical application. These issues open new avenues for designing advanced catalyst architectures that maximize atomic utilization with tunable active sites.

SACs have become excellent alternative electrocatalysts [28,29,30] to address these issues. In SACs, isolated single metal atoms are strongly stabilized on appropriate supports through strong metal–support interactions (MSIs), coordination with heteroatoms, defect anchoring, and lattice confinement. The atomic dispersion of metal species maximizes metal utilization, where every isolated metal atom can participate as an accessible active catalytic site. In addition, the coordination environment, electronic configuration, oxidation state, and adjacent atomic structures of distinct metal centers were rationally engineered. Therefore, SACs offer an excellent opportunity to establish well-defined relationships among atomic structure, electronic structure, reaction pathways, and catalytic activity/durability.

For the HER, SACs with exceptional electronic and geometric features can be used to monitor the adsorption and conversion of key reaction intermediates [31,32,33]. The adsorption free energy of H* can be optimized by monitoring the metal center and its coordination atmosphere to facilitate effective hydrogen adsorption and desorption. Water dissociation is often linked with additional kinetic restrictions in alkaline media; SACs can encourage H_2_O adsorption and cleavage via suitably designed metal–support interfaces and dual-site configurations. In addition, strong interactions between atomically dispersed single atoms and their supports can induce orbital hybridization, charge redistribution, modified electronic states, and interfacial electric fields, thus accelerating the interfacial charge transfer and enhancing the reaction energetics. The atomic-level tunability of SACs allows for the creation of synergistic active sites in which dissimilar adjacent atoms selectively endorse water adsorption, H-OH bond cleavage, hydrogen intermediate formation, and H_2_ evolution. These features make SACs effective electrocatalysts for the emerging, highly efficient HER.

**Figure 1:**
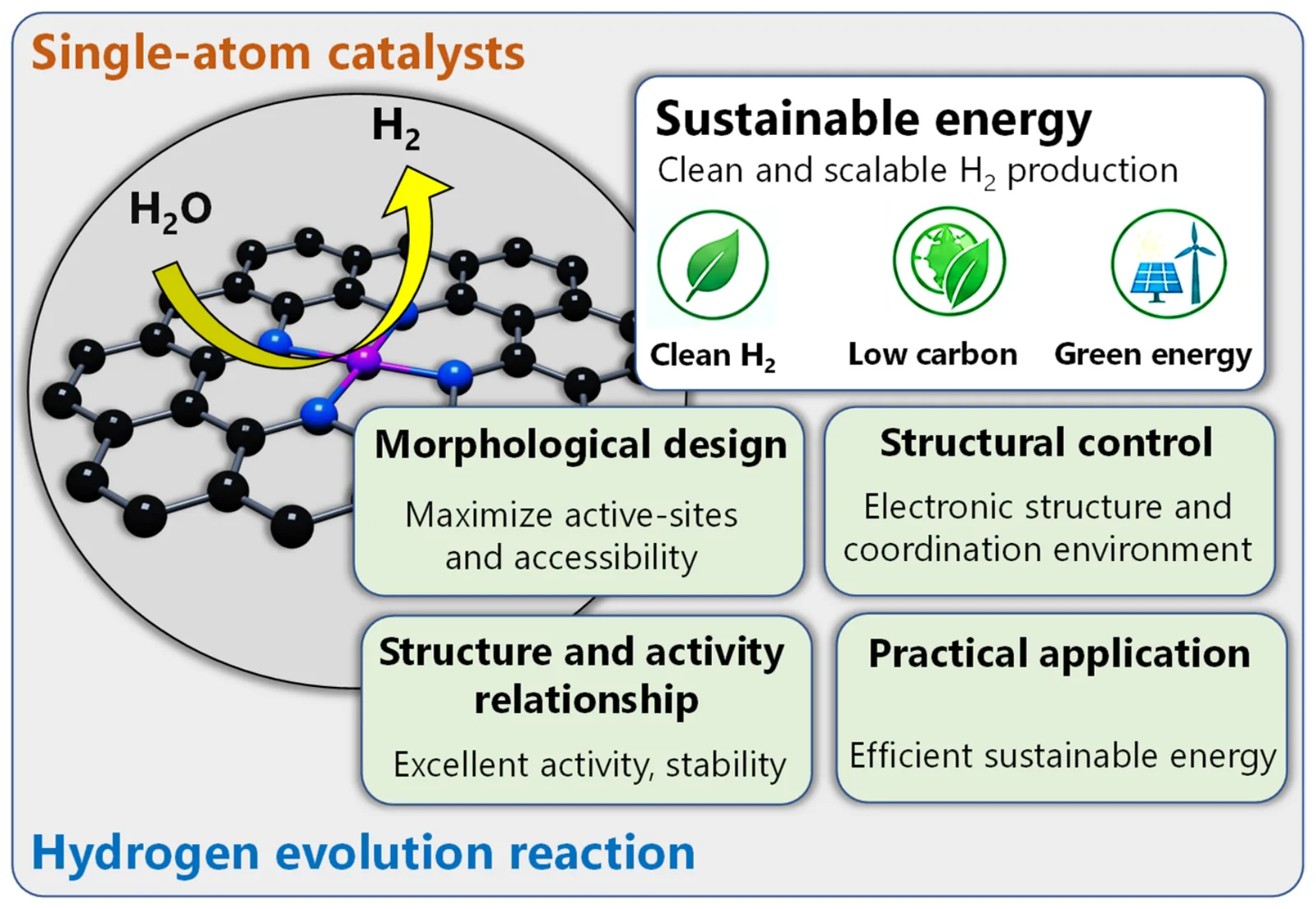
Schematic overview of SACs for HER electrocatalysis. The arrow represents water converts to hydrogen through SACs for HER.

This review provides a comprehensive overview of the promising developments in SACs for the HER, focusing on atomic-level engineering and catalytic mechanisms. We first focus on the fundamental principles and reaction mechanisms of the HER. Subsequently, the morphological engineering of abundant SAC nanostructures is discussed based on their structural features and key roles in boosting HER performance. Then, the structural characteristics of SACs are studied to clarify the roles of orbital interactions, charge redistribution, local structural asymmetry, and metal–support interactions in regulating hydrogen adsorption and HER kinetics. The catalytic performance is investigated extensively in terms of HER activity, reaction kinetics, and long-term stability. Following this, their DFT results are discussed to elucidate the mechanism underlying their outstanding catalytic performance toward the HER. Finally, key challenges associated with scalability, catalyst stability, and long-term operation at practically relevant current densities are critically discussed. Future research directions are proposed, highlighting operando characterization and data-driven approaches for the development of high-performance SACs. Finally, this review focuses on the development of a systematic pathway for the rational strategy of next-generation SACs for sustainable hydrogen production. A schematic overview is presented in Figure 1 for the recent advances in SACs for HER electrocatalysis.

## 2. Mechanistic Pathways of HER

The HER is an important electrochemical process and a key step in hydrogen production. The net reaction looks simple, 2H^+^ + 2e^−^ → H_2_ (in acidic media), but the underlying HER mechanism involves multiple sequential elementary steps governed by surface chemistry, intermediate adsorption, and interfacial charge transfer [34]. The HER process is strongly influenced by the physicochemical properties of the electrocatalyst surface, electrolyte composition, and pH of the reaction atmosphere. Therefore, a comprehensive understanding of HER kinetics and reaction pathways is crucial to the rational design of electrocatalysts under various reaction conditions.

The Tafel slope is broadly studied to examine HER kinetics and get initial understandings into the probable rate-determining step (RDS) and reaction path. The HER process usually transfers via a preliminary electrochemical hydrogen adsorption step (Volmer), followed by either electrochemical desorption (Heyrovsky) or chemical recombination (Tafel). Though distinguishing Tafel slopes of nearly 120, 40, and 30 mV dec^−1^ are conventionally related with these basic steps, their interpretation requires careful attention to be paid to hydrogen coverage, theoretical calculations, electrolyte environments, pH-dependent kinetic measurements, electrochemical impedance spectroscopy and experimental kinetics.

The HER generally proceeds via two principal mechanistic pathways: the Volmer–Heyrovsky and Volmer–Tafel mechanisms. Both pathways comprise the formation of adsorbed hydrogen intermediates (H*) on the catalyst surface, followed by their conversion into molecular H_2_. The key dissimilarity among these pathways lies in the successive reaction of the H* intermediates during H_2_ formation.

(a) Volmer–Heyrovsky Mechanism. Volmer step (electrochemical discharge) [35,36]:

*H*^+^ + *e*^−^ → *H*^∗^ (*acidic*) (1)

*H*_2_*O* + *e*^−^ → *H*^∗^ +*OH*^−^ (alkaline) (2)

Heyrovsky step (electrochemical desorption) [37,38]:

*H*^∗^ + *H*^+^ + *e*^−^ → *H*_2_ (acidic) (3)

*H*^∗^ + *H*_2_*O* + *e*^−^ → *H*_2_ + *OH*^−^ (alkaline) (4)

(b) Volmer–Tafel Mechanism. Volmer step (same as above).

Tafel steps (chemical recombination) [39]:

*H*^∗^ + *H*^∗^→ *H*_2_ (5)

The Volmer step is common to both pathways and involves the initial adsorption of a hydrogen atom onto the catalyst surface. The Heyrovsky step combines this intermediate with a proton and electron to form H_2_, whereas the Tafel step involves the recombination of two H* species. The favored pathway depends on the intrinsic properties of the catalyst, such as the hydrogen binding strength, electronic structure, electrolyte atmosphere, and reaction environment.

## 3. Morphological Influence of SACs on HER Activity

The morphology of SACs plays a key role in determining their HER performance by modulating active-site accessibility, interfacial charge transfer and mass transport [40,41,42]. Well-designed morphological architectures, such as nanosheets, nanoframes, porous structures, vacancy, core–shell, defect-rich supports and various types of morphological structures can maximize the exposure of atomically dispersed isolated-metal sites and strongly facilitate electrolyte diffusion. Thus, morphological engineering is strongly associated with several factors which directly tune the structural environments and catalytic performance. In addition, the strong interactions between isolated single atoms and the support further restrict the aggregation of particles and modify the local electronic structure. In addition, hierarchical nanoarchitectures deliver plentiful channels for reactant diffusion and H_2_ release, which help to reduce significant transport limitations during the HER. Therefore, the morphological engineering of SACs is indispensable to maximizing active-site utilization and accomplishing effective HER kinetics at various current densities.

### Engineering Morphology of SACs for HER Electrocatalysis

**Figure 2:**
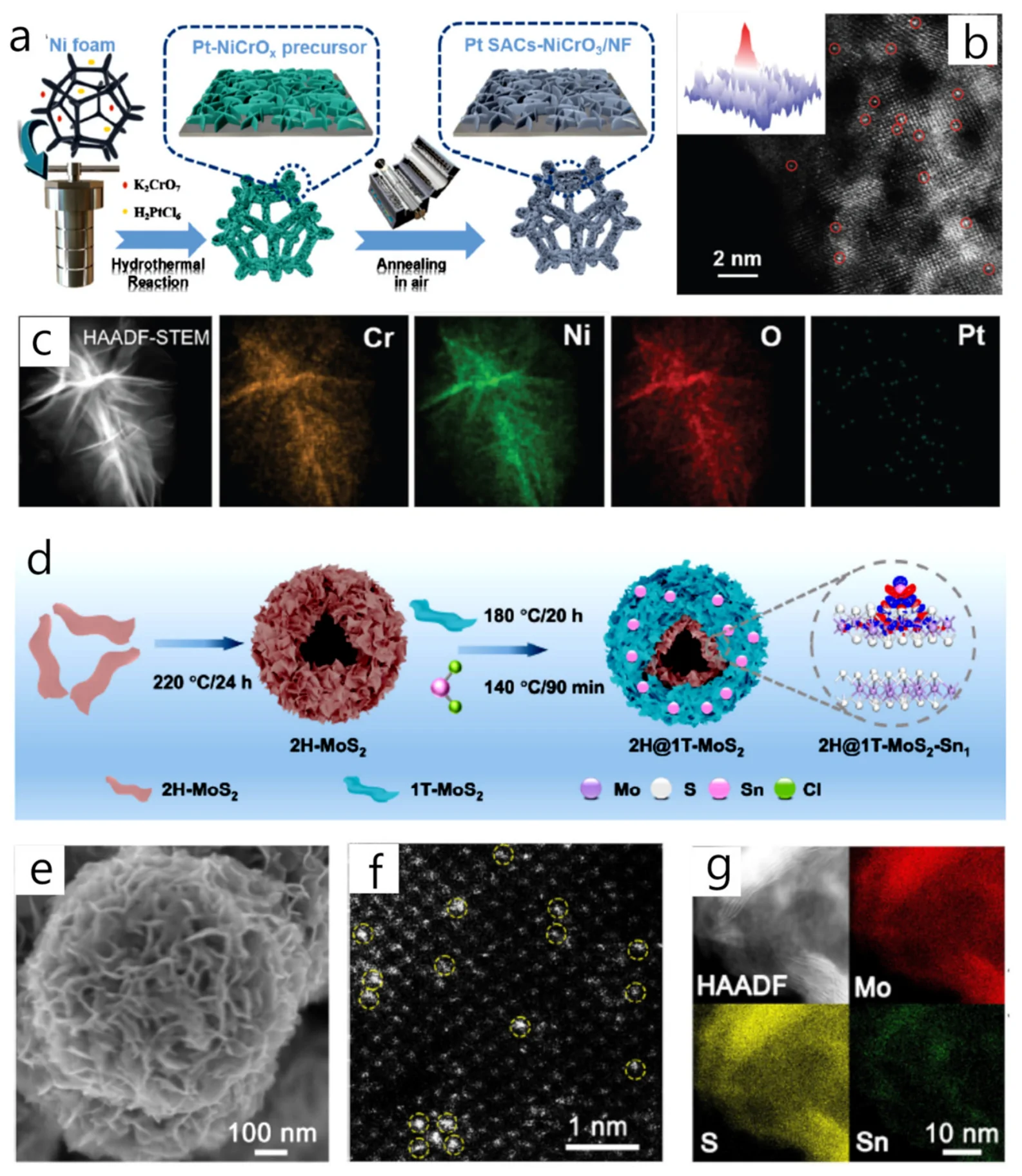
(**a**) Outline of synthesis procedure for Pt SACs anchored on nickel–chromium oxides (Pt SAC-NiCrO_3_/NF); (**b**) AC-HAADF-STEM image of the Pt SAC-NiCrO_3_/NF catalyst; (**c**) STEM-EDX images of Pt SAC-NiCrO_3_/NF catalyst. Copyright 2025, Wiley-VCH Verlag GmbH & Co. KGaA, Weinheim [43]. (**d**) Schematic of the synthesis method of the 2H@1T-MoS_2_-Sn_1_ catalyst; (**e**) scanning electron microscopy image, (**f**) AC-TEM image, and (**g**) HAADF and energy-dispersive spectroscopy (EDS) mapping of 2H@1T-MoS_2_-Sn_1_ catalyst. Copyright 2025, Wiley-VCH Verlag GmbH & Co. KGaA, Weinheim [46].

Meng et al. [43] designed an effective catalyst for the HER based on Pt SACs anchored on nickel–chromium oxides (Pt SAC-NiCrO_3_/NF). Figure 2a shows a schematic of the synthesis of the Pt SAC-NiCrO_3_/NF catalyst. The synthesis process involved a hydrothermal reaction followed by high-temperature air calcination. To explore the SAC structure, aberration-corrected (AC) high-angle annular dark-field scanning transmission electron microscopy (HAADF-STEM) images and the corresponding 3D atom-overlapping Gaussian-function fitting mappings (Figure 2b) were obtained to display the atomic-level distribution of single Pt atoms (highlighted by red circles) on the oxide support in a high-density state [44,45]. In addition, the STEM-EDX image (Figure 2c) revealed the homogeneous dispersion of the existing elements (Ni, Cr, O, and Pt) throughout the nanosheets in the Pt SAC-NiCrO_3_/NF catalyst, further confirming the successful synthesis of Pt SACs on the NiCrO_3_ support. The nanosheet structure of the Pt SAC-NiCrO_3_/NF catalyst facilitated strong metal–support interactions (MSIs) to improve the electrocatalytic HER. The strong interfacial effect modulated the electronic structure and led to charge redistribution of the metal active sites, which play a critical role in enhancing the hydrogen adsorption energetics. Thus, the rational engineering of MSIs in the Pt SAC-NiCrO_3_/NF catalyst provided an active approach to boosting HER electrocatalysts.

Jin et al. [46] reported an excellent catalyst as a hollow core–shell 2H@1T-MoS_2_-Sn_1_ nanoreactor for the acidic HER in which isolated Sn atoms were strongly anchored on the shell of the 2H@1T-MoS_2_ Mott–Schottky phase junction. Figure 2d shows a schematic synthesis diagram of the 2H@1TMoS_2_-Sn_1_ nanoreactor catalyst. The synthesis involved two key steps. First, the hydrothermal method was used to synthesize a hollow core–shell 2H@1TMoS_2_ Mott–Schottky phase junction. Next, low-temperature calcination was performed to deposit atomically dispersed Sn atoms on the 2H@1T-MoS_2_ shell. A morphological investigation was performed to elucidate the structural features of the core–shell 2H@1T-MoS_2_-Sn_1_ nanoreactor, which provided key evidence regarding catalyst morphology, surface structures, and spatial distribution of active elements. Figure 2e shows the hollow nanoflower ball-like structure of the 2H@1T-MoS_2_-Sn_1_ nanoreactor catalyst with lateral dimensions of 400–600 nm. Figure 2f shows an aberration-corrected transmission electron microscopy (AC-TEM) image which reveals bright spots on the 2H@1T-MoS_2_ surface (highlighted by yellow circles). These findings indicate the homogeneous dispersion of isolated Sn atoms in the core–shell 2H@1T-MoS_2_-Sn_1_ nanoreactor. The HAADF image and EDS analysis (Figure 2g) further supported the good dispersion of Mo, S, and Sn atoms throughout the 2H@1T-MoS_2_-Sn_1_ nanoreactor. Inductively coupled plasma mass spectrometry (ICP-MS) results confirmed that the wt% of isolated Sn atoms in the 2H@1T-MoS_2_-Sn_1_ nanoreactor was roughly 1.60%. Therefore, the core–shell 2H@1T-MoS_2_ Mott–Schottky phase junction boosted effective charge transfer. Meanwhile, the Sn–S_2_–Mo motif in the 2H@1T-MoS_2_-Sn_1_ nanoreactor greatly facilitated adsorbed H^+^ reduction and accelerated the HER kinetics.

**Figure 3:**
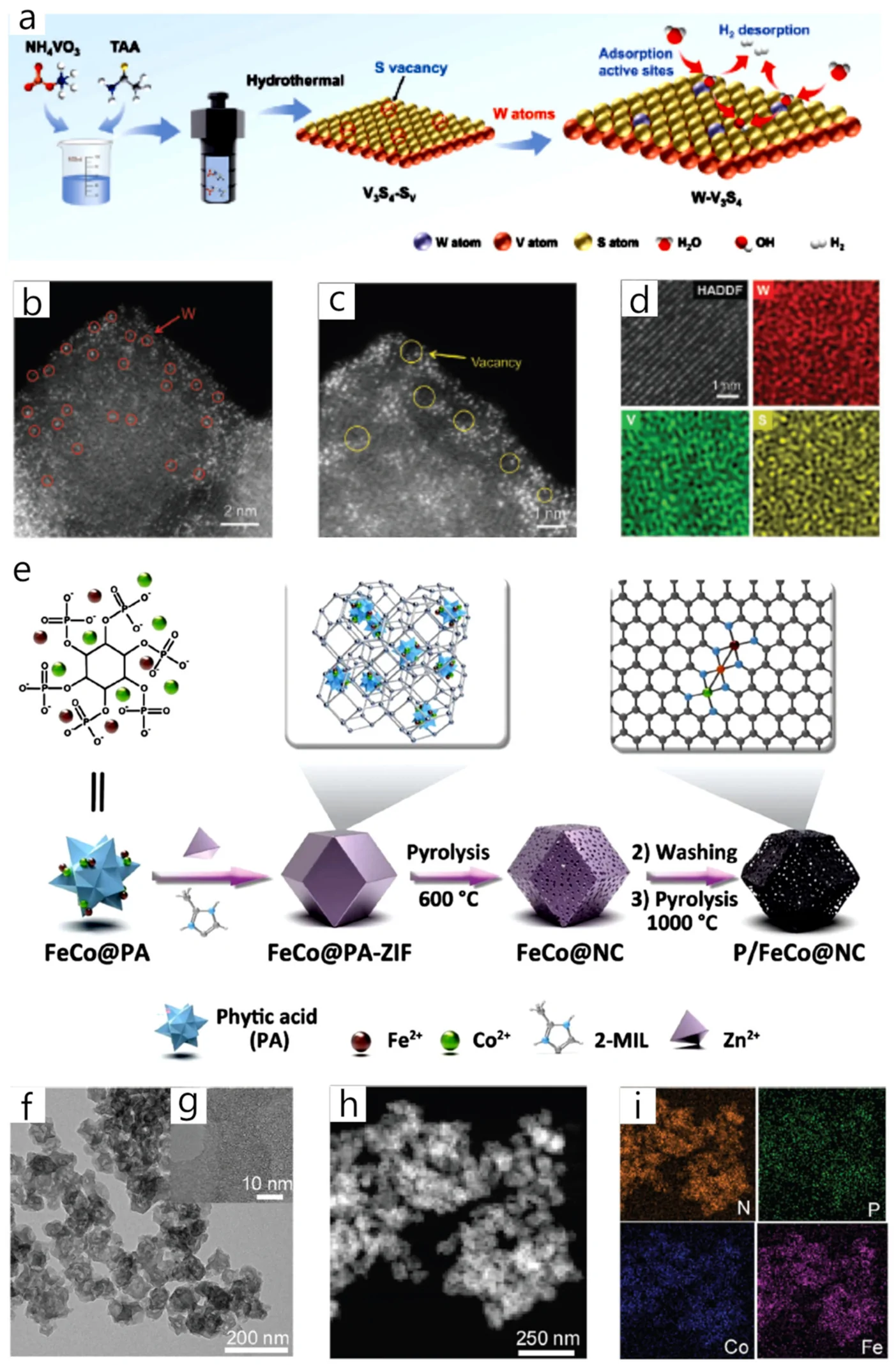
(**a**) Schematic for synthetic process of W-V_3_S_4_ catalyst; (**b**,**c**) high-resolution TEM (HRTEM) images of W-V_3_S_4_ catalyst; (**d**) HAADF-STEM image and EDS elemental mapping of W, V and S in designed W-V_3_S_4_ catalyst. Copyright 2025, Wiley-VCH Verlag GmbH & Co. KGaA, Weinheim [47]. (**e**) Schematic of synthesis of P/FeCo-NC catalyst via two-step pyrolysis treatment, (**f**) TEM image, (**g**) high-resolution TEM image, (**h**) HAADF-STEM image and (**i**) elemental mapping showing dispersion of N (orange), P (green), Co (blue) and Fe (purple). Copyright 2025, Wiley-VCH Verlag GmbH & Co. KGaA, Weinheim [51].

Xi et al. [47] designed an effective catalyst with sulfur (S) vacancy richness to anchor isolated tungsten (W) atoms onto ultrathin V_3_S_4_ nanosheets (NSs) for high-performance HER. Figure 3a shows a schematic representation of the synthesis of the atomically dispersed W single-atom (SA)-decorated V_3_S_4_ NSs. First, hydrothermal process was performed to prepare S-vacancy-rich V_3_S_4_ NSs. Subsequently, the isolated W SAs were successfully incorporated into ultrathin V_3_S_4_ NSs through in situ anchoring process. Figure 3b,c show HAADF-STEM images representing the existence of isolated W SAs and S vacancies dispersed in the V_3_S_4_ NSs. The homogeneous dispersion of isolated W SAs is clearly visible without any visible W nanoparticles or clusters on the V_3_S_4_ NSs, as highlighted by the red circles (Figure 3b). In addition, the presence of S vacancies was confirmed in the ultrathin NSs, which are marked with yellow circles (Figure 3c), and EDS mapping confirmed the homogeneous dispersion of W, V and S throughout the ultrathin V_3_S_4_ NSs (Figure 3d). Therefore, the incorporation of heteroatoms at the atomic level can tune the electronic band structure and valence states, thereby enhancing the adsorption/desorption energetics of the main reaction intermediates and enabling the total HER kinetics [48]. Consequently, defect-rich ultrathin V_3_S_4_ NSs with isolated W SAs played a key role in improving alkaline HER performance through significant MSIs and synergistic effects [49,50].

Tao et al. [51] designed a superior electrocatalyst for the high-performance HER based on phosphorus (P)-doped Fe and Co bimetallic SACs embedded on N-doped graphitic carbon (P/FeCo-NC). Figure 3e shows a schematic diagram of the synthesis of the P/FeCo-NC catalyst. Two-step pyrolysis was performed at high temperatures to obtain highly efficient P-doped SACs, as shown in Figure 3e. A morphological analysis of the P/FeCo-NC catalyst was performed using TEM to explore its structural features. Figure 3f displays the porous morphology of the P/FeCo-NC catalyst without noticeable Fe- or Co-containing nanoparticles in the high-resolution TEM (Figure 3g) and HAADF-STEM images (Figure 3h). In addition, EDS color mapping revealed the homogeneous dispersion of N, P, Fe and Co in the carbon support of the bimetallic P/FeCo-NC catalyst (Figure 3i). These findings provide important insights into the role of doped P in bimetallic SACs for enhancing HER performance and offer a potential pathway for designing effective catalysts.

## 4. Structural Depiction of SACs for Exploring HER Performance

Structural characterization is important to identify the atomic structures of SACs associated with improved HER performance [52,53,54]. X-ray diffraction (XRD) analysis was used to identify the crystalline phase and explore the nature of the metal nanoparticles or secondary phases. X-ray photoelectron spectroscopy (XPS) disclosed the oxidation states and electronic atmospheres of isolated active metal sites. X-ray absorption near-edge structure (XANES) analysis provided evidence of the electronic structure and valence state, whereas extended X-ray absorption fine structure (EXAFS) analysis elucidated the bond distances, local coordination environment, and coordination numbers of SA sites. Brunauer–Emmett–Teller (BET) analysis was used to evaluate the specific surface area and pore characteristics which influence the active-site exposure and mass transport. Together, these structural analyses established structure–activity relationships of the developed various morphological architectures and explained the causes of the improved HER kinetics of various designed catalysts and their comparative aspects.

**Figure 4:**
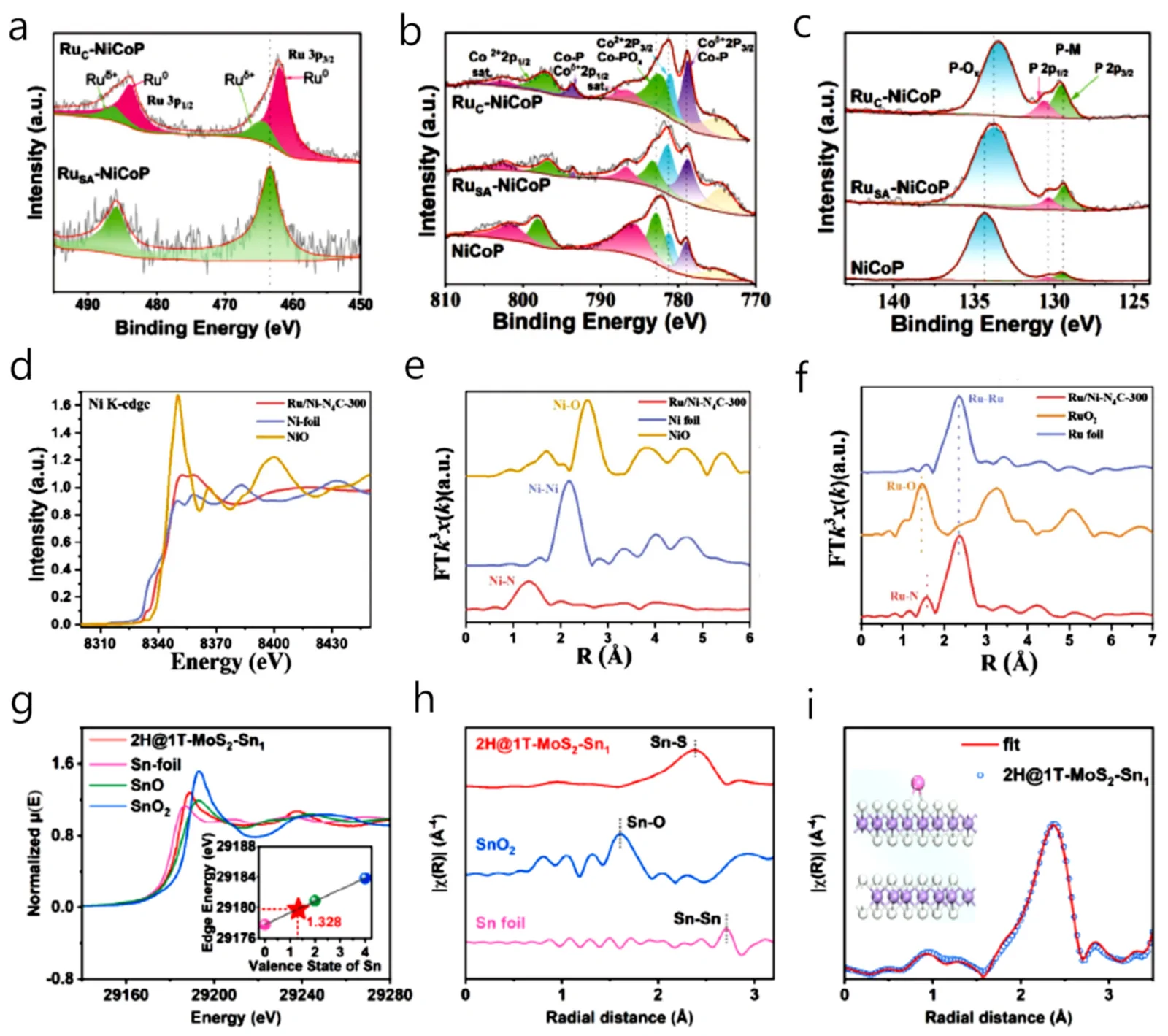
High-resolution XPS spectra of (**a**) Ru 3*p*, (**b**) Co 2*p*, and (**c**) P 2*p* for Ru_C_-NiCoP, Ru_SA_-NiCoP, and NiCoP catalysts. Copyright 2025, Wiley-VCH Verlag GmbH & Co. KGaA, Weinheim [55]. (**d**) Ni K-edge XANES spectra and (**e**) *k*_3_-weighted Fourier-transformed (FT) EXAFS spectra of Ru/Ni-N_4_C-300 catalyst, with Ni foil and NiO as reference catalysts. (**f**) *k*_3_-Weighted FT-EXAFS spectral analysis of Ru/Ni-N_4_C-300, Ru foil, and RuO_2_ catalysts. Copyright 2025, Wiley-VCH Verlag GmbH & Co. KGaA, Weinheim [65]; structural evidence of the 2H@1T-MoS_2_-Sn_1_ nanoreactor. (**g**) Normalized K-edge XANES of Sn, (**h**) EXAFS spectral analysis of Sn K-edge and (**i**) EXAFS spectral fitting curves of Sn in R-space. Copyright 2025, Wiley-VCH Verlag GmbH & Co. KGaA, Weinheim [46].

Liu et al. [55] designed a superior single-atom electrocatalyst consisting of ruthenium SAs (Ru_SA_) immobilized onto NiCoP nanosheets to enhance electrocatalytic activity for the HER. Structure is a key physicochemical parameter to obtain insights into catalysts and understand their effects on catalytic performance. XPS analysis was performed to explore the surface chemical bonding states of the Ru_C_-NiCoP, Ru_SA_-NiCoP and NiCoP catalysts. The Ru 3*p* spectrum of the Ru_SA_-NiCoP catalyst (Figure 4a) displayed two main binding energy peaks at ~463.38 and ~486.08 eV, indicating the existence of a positively charged valence state of isolated Ru atoms [56]. In contrast, the Ru 3*p* spectrum of the Ru_C_-NiCoP catalyst showed two prominent peaks at ~461.98 and ~484.08 eV. This result supports the presence of metallic Ru atoms in Ru_C_ species [57,58,59]. The appearance of a characteristic peak at ~280.18 eV in the Ru 3*d* spectrum of the Ru_C_-NiCoP catalyst was assigned to Ru 3*d*_5/2_ of Ru_0_, suggesting the formation of Ru-Ru bonds within the Ru_C_ species [60,61]. Furthermore, the Ru 3*d* spectrum of the Ru_C_-NiCoP catalyst displayed significantly greater peak intensity at ~282.28 eV than the Ru_SA_-NiCoP catalyst, due to the Ru 3d_5/2_ of Ru*δ*+. This intensity enhancement indicates stronger electronic interactions among the Ru_C_ species and the NiCoP support. Figure 4b demonstrates the high resolution of the Co 2*p* binding energy spectra. The NiCoP spectrum displays two major binding energy peaks at ~778.98 and ~793.88 eV, assigned to 2*p*_3/2_ and 2*p*_1/2_, respectively, of Co*δ*+ (*δ* close to 0) in the Co-P bond [62]. In addition, the appearance of different peaks at ~781.28, ~782.88 and ~798.18 eV, accompanied by broad satellite peaks at ~785.88 and ~801.68 eV, was attributed to the 2*p*_3/2_ and 2*p*_1/2_ of Co^2+^ in Co-POx (resulting from the surface oxidation of NiCoP) [63,64]. Following Ru_SA_ electrodeposition, the Co^2+^ 2*p*_3/2_ spin–orbit peak of the Ru_SA_-NiCoP catalyst shifted toward higher binding energy, while the Co*^*δ*^*^+^ 2*p*_3/2_ peak displayed an insignificant shift toward lower binding energy. This finding confirmed an electron transfer from Ru_SA_ species to surrounding Co atoms, which helped to modulate the interfacial electronic distribution state and enable interfacial charge transfer across the Ru_SA_-NiCoP interface. Following Ru_C_ electrodeposition of the Ru_C_-NiCoP catalyst, the higher, broadened peak of Co^2+^ 2*p*_3/2_ appeared at ~782.88, whereas the peak of Co*^*δ*^*^+^ 2*p*_3/2_ revealed a significant intensity enhancement with a small negative shift. Figure 4c displays the high-resolution spectral analysis for P 2*p*. Two prominent peaks appeared at ~129.38 and ~130.38 eV in the NiCoP spectrum and were attributed to the P 2*p*_3/2_ and P 2*p*_1/2_ spin orbitals in metal phosphide P-M bonds, respectively [56]. The appearance of a broad peak at ~134.38 eV was due to the oxidized P species derived from NiCoP nanosheet surface oxidation. Following electrodeposition of Ru_C_ or Ru_SA_, a noticeable enhancement in the peak intensities was found, corresponding to P 2*p*_3/2_ and P 2*p*_1/2_, which demonstrates strengthened P-M bonding affected by either Ru_C_ or Ru_SA_, respectively. Liu et al. [55] performed XRD analysis in their study. The diffraction patterns of Ru_C_–NiCoP and Ru_SA_–NiCoP closely looked like that of NiCoP without any noticeable diffraction peaks attributable to crystalline Ru. This observation supports that Ru_C_ particles are adequately insignificant to remain below the detection border of XRD analysis.

Zhang et al. [65] synthesized SA Ni-N_4_ site-supported ruthenium nanoclusters (Ru NCs) for the enhancement in HER performance. XANES results were studied to explore the local atomic coordination surroundings of Ni and Ru in the Ru/Ni-N_4_C-300 catalyst. The Ru/Ni-N_4_C-300 catalyst showed white-line intensity (Figure 4d) within the well-defined range of Ni foil and NiO, which suggests an oxidation valence state structure of Ni*δ*+ (0 < *δ* < 2). This oxidation state is characteristic of dispersed isolated Ni atoms in a square-planar coordination atmosphere within the carbon-based substrate comprising N donor groups [66]. In addition, the Ru/Ni-N_4_C-300 catalyst showed a low-intensity pre-edge peak at ~8340 eV, ascribed to the dipole-allowed transitions from 1*s* to 4*p*_z_ orbitals. This finding supports the existence of square-planar Ni-N_4_ units with disordered D_4h_ symmetry [67]. Further, a distinctive Fourier-transformed (FT) XAFS peak for Ni-N coordination appeared at ~1.5 Å, confirming the absence of Ni(0) and NiO NPs in the Ru/Ni-N_4_C-300 catalyst (Figure 4e) [66]. This observation confirms the existence of isolated Ni SAs within the Ru/Ni-N_4_C-300 catalyst support material [68]. FT-EXAFS Ru K-edge analysis was performed to probe the electronic structure of the Ru/Ni-N_4_C-300 catalyst. The appearance of the prominent peak at ~2.3 Å indicates the Ru-Ru scattering of the Ru NCs (Figure 4f). Another prominent peak at ~1.4 Å was assigned to Ru-N bonds, supporting the coordination of Ru species with N atoms. This interaction modulated the electronic structure of the Ru nanoclusters, which may have contributed to their improved catalytic performance [69]. Therefore, modulating the electron density of Ru nanoclusters through isolated Ni-N_4_ sites allows for the expansion of stable, alkaline HER catalysts with potential for industrially relevant operating conditions.

Jin et al. [46] reported an efficient electrocatalyst as a hollow core–shell 2H@1T-MoS_2_-Sn_1_ nanoreactor for high-performance HER under acidic conditions. XANES and EXAFS analyses were performed to analyze the absorption edges and local microstructure of the 2H@1T-MoS_2_-Sn_1_ nanoreactor. The absorption edge of the 2H@1T-MoS_2_-Sn_1_ nanoreactor was observed between the absorption edges of Sn foil and SnO (Figure 4g). This finding confirms that the oxidation state of Sn was between 0 and +2 in the 2H@1T-MoS_2_-Sn_1_ nanoreactor [70]. The FT-EXAFS spectrum analysis also confirmed (Figure 4h) that Sn was mainly present as isolated SA sites rather than as SnO_2_ or Sn nanoparticles. The fitting results achieved from the correlation between the K-space and R-space data imply that the isolated Sn atoms were coordinated with the S_2_-Mo structure, forming a distinct Sn-S_2_-Mo motif. Moreover, the synchrotron radiation photoemission spectroscopy (SRPES) results from 2H@1T-MoS_2_-Sn_1_ further indicated the development of Sn-S bonds. Figure 4i shows that the bond length of S-Sn was ~2.556 Å in the 2H@1T-MoS_2_-Sn_1_ nanoreactor. This exceptional structural conformation encourages effective water dissociation and improves H* adsorption, thus facilitating the HER process and overall electrocatalytic performance.

Meng et al. [43] developed an effective Pt SAC-NiCrO_3_/NF catalyst for the HER. XAFS analysis was done to explore the coordination information and electronic structure of Pt SACs on the Pt SAC-NiCrO_3_/NF catalyst. The normalized Pt L3-edge spectra and the partially enlarged diagram showed the threshold value (E_0_) of Pt SAC-NiCrO_3_/NF of 11,568.5 eV, which was greater than that of Pt foil (11,567.2 eV) and adjacent to that of PtO_2_ (11,569.0 eV). This result confirmed its high-valence state related with lesser transition energy from 1s to the outermost shell orbitals. Besides, EXAFS disclosed the local coordination surroundings of Pt species. In the Pt L3-edge EXAFS spectra, the appearance of a prominent peak at ~1.65 Å aligned with the Pt-O coordination first shell, which is comparable to PtO_2_. Thus, XAFS results established that isolated single Pt is atomically distributed within the oxide phase and coordinated via oxygen bridges. In addition, the XPS result displayed three typical peaks at ~530.0~530.8, and ~531.6 eV for the O 1s spectrum, which match metal–oxygen (M-O) bonds, oxygen vacancies and surface-adsorbed oxygen functional groups, respectively. The variation in the peak intensity ratio of M-O bonds and oxygen vacancies indicates differences in the Pt–O bridging coordination atmosphere. These deviations are meticulously related with metal–support interaction (MSI) effect. Overall, the integration of atomically dispersed Pt single atoms onto the NiCrO_3_/NF support through coordination of oxygen bridges generates advantageous metal–support interaction, which plays a critical role in developing synergistic co-catalytic effects among the two active components and could be explored as a suitable microenvironment for boosting HER activity.

Jin et al. [46] reported an excellent 2H@1T-MoS_2_-Sn_1_ nanoreactor catalyst for the HER. XANES and EXAFS analyses were done to expose the local microstructure of a 2H@1T-MoS_2_-Sn_1_ nanoreactor along with Sn foil, SnO and SnO_2_. The absorption edge of 2H@1T-MoS_2_-Sn_1_ was located among that of Sn foil and SnO. This result confirmed the valence oxidation state of Sn: between 0 and +2. In addition, FT-EXAFS spectra confirmed that Sn was found mostly as isolated single atoms rather than as SnO_2_ and Sn nanoparticles. Further, EXAFS fitting results supported that Sn atoms coordinate with the S_2_-Mo structure to make the Sn–S_2_–Mo motif in the 2H@1T-MoS_2_-Sn_1_ nanoreactor. Thus, the Sn-S_2_-Mo motif enables the lessening of adsorbed H^+^, greatly enhancing HER kinetics. An electron density difference (EDD) study further disclosed accumulation of charge around the isolated atomically dispersed Sn single atoms and charge reduction around the Mo atoms. This finding designates that integration of Sn and encourages electron reorganization within the 2H@1T-MoS_2_-Sn_1_ nanoreactor. Thus, the electrons are moved directionally from Mo to S and then to Sn. This facilitates the formation of electron-rich Sn sites with strong reduction capability, thereby assisting in H^+^ reduction during the HER.

Xi et al. [47] designed S vacancy richness to anchor isolated W atoms onto ultrathin V_3_S_4_ NSs for the HER. XAS was studied to explore the coordination atmosphere and atomic-level electronic structure of the W-V_3_S_4_ catalyst. The W L3-edge XANES spectra of W-V_3_S_4_ (10,202 eV) designated a positively charged W oxidation state between W^4+^ and W^6+^ by comparison with W foil, WO_2_ and WO_3_. The pre-edge white line intensity of W-V_3_S_4_ with a dominant peak at ~10,210 eV was little higher compared to WO_2_, which suggests a highly localized W 5d electronic state. FT-EXAFS and WT spectra allow for high-resolution investigation in both k and R spaces. The W-V_3_S_4_ catalyst showed two distinguished coordination peaks at ~1.63 and ~2.73 Å, which were associated with the first coordination shell of W-O and W-V. The existence of W-O bonds indicated oxidation on the catalyst surface, whereas the nonappearance of the W-W (~2.64 Å) bonds confirmed the development of atomically dispersed isolated W SAs. Hence, incorporating isolated W SAs efficiently directed to charge density relocation and improved the adsorption/desorption of the intermediates, boosting HER catalytic activity.

Tao et al. [51] designed a P/FeCo-NC electrocatalyst for the HER. XANES and EXAFS analyses were done to explore the coordination environments of metals in the P/FeCo-NC catalyst along with standard Fe foil, FeO and Fe_2_O_3_. Fe K-edge XANES spectra confirmed that the valence oxidation state of Fe species in the P/FeCo-NC catalyst was higher than 2+. Co K-edge XANES spectra confirmed the oxidation state of Co with the average valence oxidation of +2. In the Fe K-edge FT EXAFS R-space, the spectrum displayed single dominant peak at ~1.5 Å for P/FeCo-NC, attributed to the first coordination shell of the Fe-N bond. In addition, the absence of the Fe-Fe coordination peak at ~2.2 Å compared with Fe foil confirmed that the Fe species in P/FeCo-NC were atomically dispersed. The atomically dispersed single-Co-atom site in P/FeCo-NC was further established by the FT Co K-edge EXAFS spectra. The Co-N peak was moved down by ~0.05 Å in P/FeCo-NC compared to the Co-N peak at single-Co sites (~1.5 Å). This result suggested that the electron density around the central single Co atoms was modulated by the integration of P atoms. The EXAFS fitting confirmed that four N were situated in the first coordination environment whereas P existed in the second coordination sphere. Besides, DFT analysis exposed that P incorporation greatly influences Fe sites’ electronic structure. The P bridge among Fe and Co sites improves the hydrogen adsorption energy by controlling orbital hybridization and electronic interactions. The Fe-P-Co site showed the maximum HER activity. This improved activity is ascribed to the synergistic electronic modulation by P and Co, which results in the finest hydrogen binding energy.

## 5. Electrochemical and Theoretical Investigation of SACs Toward HER

Electrochemical and theoretical analyses are crucial to determining the efficiency of SACs in the HER [71,72,73,74,75]. Electrochemical measurements [76], including overpotential, Tafel slope, exchange current density, electrochemical impedance spectroscopy (EIS), and long-term stability provide insights into HER kinetics and charge-transfer processes. DFT calculations support elucidation of the electronic structure, hydrogen adsorption energetics, reaction pathways and active-site characteristics. Together, these analyses provide fundamental insights into the catalytic mechanisms of SACs in the HER and expose the possible reasons behind the comparative catalytic performance.

**Figure 5:**
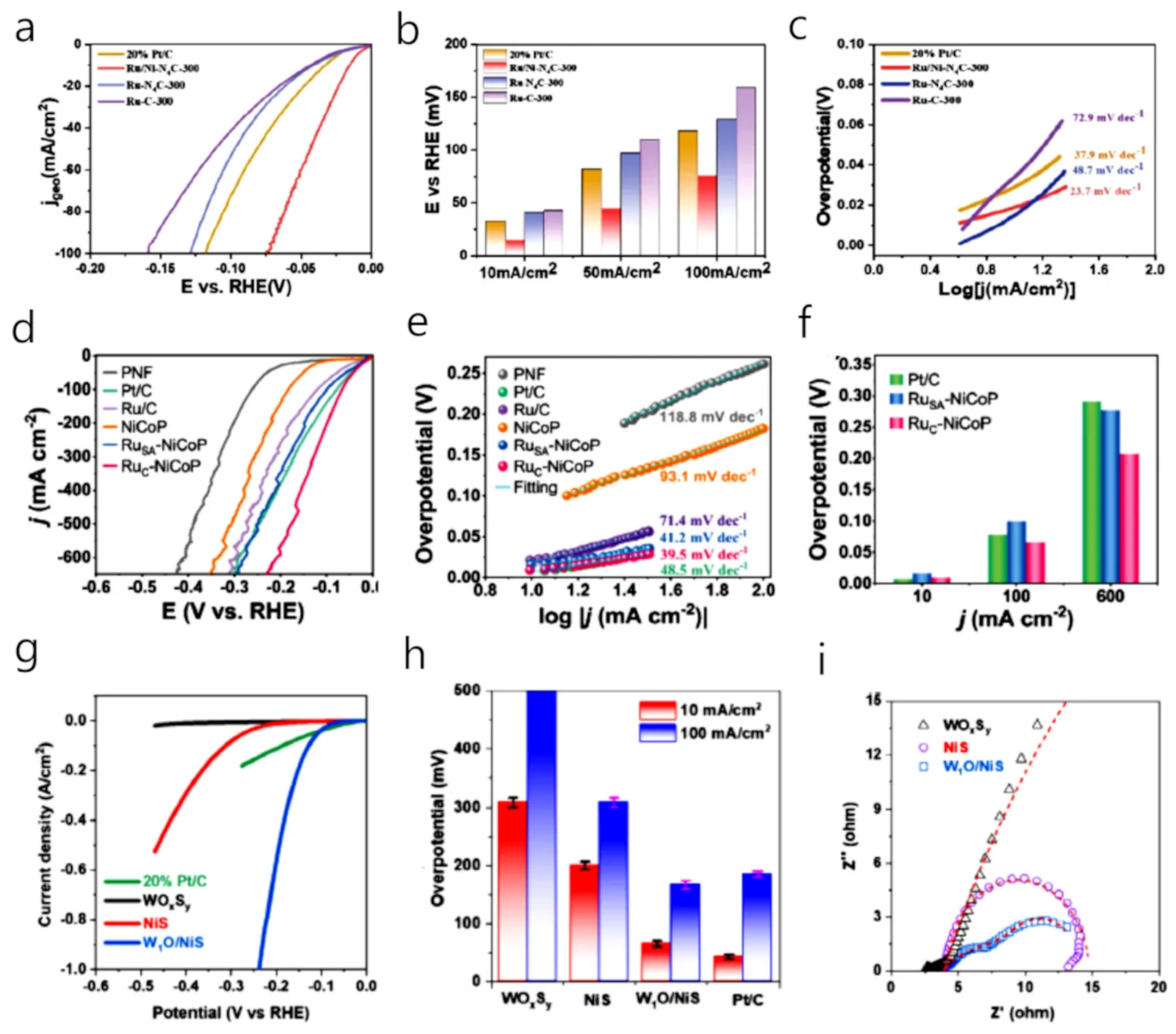
Electrocatalytic analysis of 20% Pt/C and numerous Ru-based HER catalysts in 1.0 M KOH electrolyte: (**a**) lattice vacancy site (LVS) polarization; (**b**) overpotential values at current densities of 10, 50 and 100 mA cm^−2^, respectively; (**c**) Tafel plot. Copyright 2025, Wiley-VCH Verlag GmbH & Co. KGaA, Weinheim [65]. Electrochemical studies: (**d**) LSV analysis and (**e**) Tafel plot for PtNiFe (PNF), Pt/C, Ru/C, NiCoP, Ru_SA_-NiCoP and RuC-NiCoP catalysts in 1.0 M KOH for HER; (**f**) comparison results of HER overpotentials at 10, 100 and 600 mA cm^−2^ for NiCoP, Ru_SA_-NiCoP and Ru_C_-NiCoP catalysts. Copyright 2025, Wiley-VCH Verlag GmbH & Co. KGaA, Weinheim [55]. HER performance: (**g**) HER polarization plots with iR (voltage drop) correction for NiS, WO_x_S_y_, W_1_O/NiS and Pt/C catalysts; (**h**) comparison of the overpotentials for NiS, WO_x_S_y_, W_1_O/NiS and Pt/C catalysts at various current densities; (**i**) EIS Nyquist curves for dissimilar catalysts at an overpotential of 100 mV. Copyright 2025, Wiley-VCH Verlag GmbH & Co. KGaA, Weinheim [79].

Atomically dispersed Ni-N_4_ sites on ruthenium nanoclusters (Ru NCs) were synthesized as superior electrocatalysts with promising HER performance by Zhang’s group [65]. The electrochemical HER performance of Ru/Ni-N_4_C-300 was investigated in an alkaline electrolyte solution. Linear sweep voltammetry (LSV) analysis (Figure 5a) showed that the Ru/Ni-N_4_C-300 catalyst exhibited the highest current density within the measured potential region among all the catalysts. The Ru/Ni-N_4_C-300 catalyst showed outstanding HER activity with a very low overpotential of ~15.0 mV at a current density of 10 mA cm^−2^. This result is far better than those of Ru-N_4_C-300 (~43.2 mV) and 20% Pt/C (~33.0 mV) (Figure 5b). In addition, the Ru/Ni-N_4_C-300 catalyst delivered a lower Tafel slope of ~23.7 mV dec^−1^ compared to commercial 20% Pt/C (~37.9 mV dec^−1^) (Figure 5c). This finding indicates faster HER kinetics for the Ru/Ni-N_4_C-300 catalyst [77,78], following the Volmer–Tafel mechanism. Interestingly, the values for the Tafel slopes of the other catalysts without single Ni atoms were higher (~72.9 mV dec^−1^ for the Ru-C-300 catalyst and ~48.7 mV dec^−1^ for the Ru-N_4_C-300 catalyst) compared to the Ru/Ni-N_4_C-300 catalyst. This observation directly confirms the significant effect of the atomically dispersed Ni single atoms on the Ru performance of Ru NCs. Furthermore, in an anion exchange membrane water electrolyzer (AEMWE), Ru/Ni-N_4_C-300 operated stably for over 1370 h at 500 mA cm^−2^, indicating its superiority for industrialization applications.

Highly active ruthenium single atoms (Ru_SA_) immobilized onto NiCoP were prepared to boost its bifunctional electrocatalytic activity in the HER by Liu et al. [55] in their study. The electrocatalytic performance of the catalysts was studied for the HER in 1.0 M KOH saturated with Ar. Figure 5d shows the LSV analysis (vs. RHE) curves without IR compensation. The NiCoP catalyst displayed significantly lower catalytic performance than commercial Pt/C and Ru/C catalysts. Nevertheless, an outstanding improvement in HER performance was achieved upon the electrodeposition of Ru_SA_ species onto the NiCoP surface. Thus, Ru_SA_ incorporation played a crucial role in driving the catalytic performance to the next level. Figure 5e shows the fitted Tafel slope, and the Ru_C_-NiCoP held the most promising Tafel slope value (~39.5 mV dec^−1^) among all the catalysts. The Ru_C_-NiCoP catalyst (Figure 5f) showed remarkably low overpotentials of ~10, ~66, and ~207 mV to complete H_2_ evolution current densities (j_HER_) of ~10, ~100 and ~600 mA cm^−2^, respectively. These values are lower than those obtained for commercial Pt/C catalysts (~11, ~79 and ~290 mV, respectively). This study provides a feasible strategy for the development of practical, energy-efficient, and sustainable hydrogen production technologies.

Oxygen-coordinated W SAs anchored on nickel sulfide slabs (W_1_O/NiS) were designed as efficient electrocatalysts for the HER [79]. The HER performance of the W_1_O/NiS catalysts was analyzed in an Ar-saturated 1 M KOH electrolyte solution using a conventional three-electrode system. The LSV analysis (Figure 5g,h) showed that the W_1_O/NiS catalyst provided significantly higher catalytic performance than NiS, WO_x_S_y_ and commercial Pt/C catalysts under high current (1000 mA cm^−2^). In addition, W_1_O/NiS required a negligible overpotential of ~67 mV at the current density of 10 mA cm^−2^, which is far lower than that of NiS (~196 mV) or WO_x_S_y_ (~395 mV). Furthermore, the W_1_O/NiS catalyst upheld excellent performance with an overpotential of ~166 mV at a current density of 100 mA cm^−2^, which is even better than that of the commercial 20% Pt/C catalyst (~184 mV). These findings highlight its extraordinary electrocatalytic efficacy for the alkaline HER. Additionally, EIS analyses revealed that the W_1_O/NiS catalyst displayed very low charge-transfer resistance (R_ct_ = 6.97 Ω) compared to the NiS (10.66 Ω) and WO_x_S_y_ (109.2 Ω) catalysts (Figure 5i). The EIS plot showed a smaller Nyquist semicircle for W_1_O/NiS, demonstrating lower charge-transfer resistance, increased electrochemically accessible surface area, improved electrode–electrolyte contact and more beneficial interfacial electrochemical kinetics [80]. This enhancement shows improved HER kinetics related to oxygen-coordinated isolated W single atoms and their modulation of the interfacial H_2_O structure.

**Figure 6:**
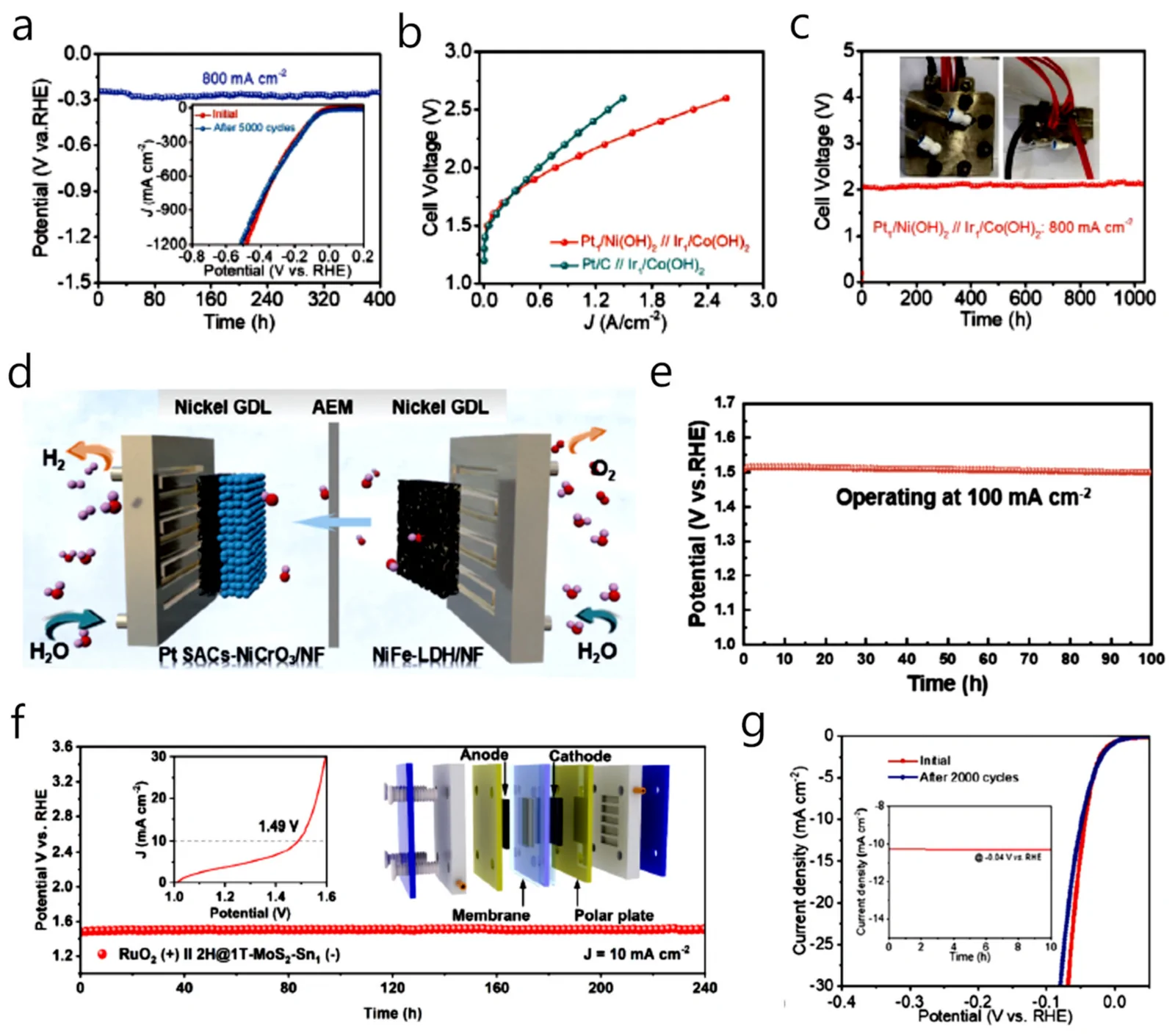
(**a**) Prolonged stability of Pt_1_/Ni(OH)_2_ during HER. Inset shows LSV results before and after CV cycles. (**b**) LSV analysis of AWE device; (**c**) stability results for AWE device. Insets show photographs of AWE device under working conditions. Copyright 2025, Wiley-VCH Verlag GmbH & Co. KGaA, Weinheim [81]. (**d**) Schematic of device assembled with Pt SAC-NiCrO_3_/NF as cathode and nickel–iron hydroxide (NiFeO_x_H_y_/NF) as anode; (**e**) chronopotentiometric analysis of Pt SAC-NiCrO_3_/NF∥NiFeO_x_H_y_/NF system at 100 mA cm^−2^. Copyright 2025, Wiley-VCH Verlag GmbH & Co. KGaA, Weinheim [43]. (**f**) Proton exchange membrane (PEM) result analysis (10 mA cm^−2^) using 2H@1T-MoS_2_-Sn_1_ nanoreactor and commercial RuO_2_ catalyst in PEM electrolyzer. Inset shows overall water-splitting polarization curve. Copyright 2025, Wiley-VCH Verlag GmbH & Co. KGaA, Weinheim [46]. (**g**) Polarization results of P/FeCo-NC electrocatalyst before and after 2000 CV cycles. Copyright 2025, Wiley-VCH Verlag GmbH & Co. KGaA, Weinheim [51].

An efficient HER catalyst consisting of atomically dispersed single Pt atoms confined within a Ni(OH)_2_ nanosheet lattice [(Pt_1_/Ni(OH)_2_)] where the Pt sites were electron-deficient was synthesized by Cao’s group for the HER [81]. Flowing anion water electrolyzer (AWE) tests were done at 80 °C in 30% KOH solution to explore the catalyst’s potential for large-scale application. Furthermore, the long-term durability of the Pt_1_/Ni(OH)_2_ catalyst under high current density is crucial to assessing its practical viability. Figure 6a shows the chronopotentiometry results and accelerated linear potential sweep analysis. Interestingly, the Pt_1_/Ni(OH)_2_ catalyst showed exceptional long-term durability at an industrially relevant current density of 800 mA cm^−2^, with only an insignificant variation in the potential required to withstand 800 mA cm^−2^ after 400 h of continuous operation. In addition, the LSV analysis of Pt_1_/Ni(OH)_2_ during the HER showed no noticeable degradation after 5000 cycles. Therefore, the lattice-confined Pt_1_/Ni(OH)_2_ catalyst displayed excellent stability under high current density owing to the confinement effect and may be applied in future industrial water splitting. For practical application, an AWE device was fabricated and studied for its long-term stability under industrial conditions. The Pt_1_/Ni(OH)_2_//Ir_1_/Co(OH)_2_ system (Figure 6b) required only ~1.6 V to achieve a current density of ~100 mA cm^−2^. More importantly, the Pt_1_/Ni(OH)_2_//Ir_1_/Co(OH)_2_ system attained a remarkably high current density of ~2250 mA cm^−2^ at a cell voltage of only 2.5 V in an AWE device. However, the Pt/C//Ir_1_/Co(OH)_2_ system reached a current density of only ~1332 mA cm^−2^ at 2.5 V. Consequently, the Pt_1_/Ni(OH)_2_//Ir_1_/Co(OH)_2_ system established superior overall water-splitting performance compared with the Pt/C//Ir_1_/Co(OH)_2_ system under the same cell voltage. In addition, the Pt_1_/Ni(OH)_2_//Ir_1_/Co(OH)_2_ system delivered notable long-term stability for 1030 h under the large current density of 800 mA cm^−2^, with insignificant reduction in the corresponding cell potential after 1030 h (Figure 6c). Therefore, the lattice-confined Pt_1_/Ni(OH)_2_ is a promising HER electrocatalyst.

An effective HER catalyst consisting of atomically dispersed Pt SACs anchored on Ni-Cr oxides (Pt SAC-NiCrO_3_/NF) was systematically studied for effective HER by Meng et al. [43]. To explore the practical viability of the catalyst, AEMWE was developed using Pt SAC-NiCrO_3_/NF as the cathode, coupled with a promising OER catalyst of nickel–iron hydroxide (NiFeO_x_H_y_/NF) as the anode (Figure 6d). This electrolyzer provided a continuous current density of 100 mA cm^−2^ at a low cell voltage of 1.51 V at room temperature in 1.0 M KOH electrolyte. This finding confirmed its superiority over the commercial Pt/C∥RuO_2_ system. Pt SACs–NiCrO_3_/NF showed a low Pt loading of ~0.21 mg_Pt_ cm^−2^ with excellent mass activity of ~1.41 A mg_Pt_^−1^ at a cell voltage of 1.7 V. Furthermore, the Pt SAC-NiCrO_3_/NF∥NiFeO_x_H_y_/NF system delivered remarkable durability (100 h of operation) at a current density of 100 mA cm^−2^ with negligible reduction (Figure 6e). In addition, the Faraday efficiency (FE) of cathodic hydrogen production was nearly 100%, as determined by gas chromatography, indicating the high efficiency of the overall water electrolysis system. Collectively, these advantages indicate the potential of the Pt SAC-NiCrO_3_/NF electrocatalyst for practical water electrolysis for efficient, sustainable green hydrogen production. Further, the characterizations studies after the stability test exhibited that the morphology, chemical composition and crystalline property of the designed catalyst persisted integrally, which strongly supported its excellent electrochemical stability during long-term cycling.

Jin’s group [46] developed a hollow core–shell 2H@1T-MoS_2_-Sn_1_ nanoreactor as an efficient electrocatalyst for the acidic HER. A proton exchange membrane (PEM) electrolyzer was assembled by integrating the 2H@1T-MoS_2_-Sn_1_ nanoreactor as a cathodic HER catalyst and commercial RuO_2_ as an anodic OER catalyst. The battery voltage of the 2H@1T-MoS_2_-Sn_1_ PEM electrolyzer was found to deliver a cell voltage of 1.49 V at 10 mA cm^−2^ (Figure 6f, inset), and no noteworthy variation in overpotential was observed during a 240 h test at 10 mA cm^−2^ (Figure 6f). This finding creates directions for large-scale future research. These results collectively establish the long-term durability of the 2H@1T-MoS_2_-Sn_1_ nanoreactor. Besides, post-analysis characterization after the cycling reaction was done to explore the impact of the 2H@1TMoS_2_-Sn_1_ nanoreactor. These post-treatment analyses together validated the excellent catalytic behavior of the designed nanoreactor.

Tao et al. [51] synthesized P-doped Fe and Co bimetallic SACs embedded in N-doped graphitic carbon (P/FeCo-NC) as active catalysts for the HER. The time-dependent current density curve (insert in Figure 6g) of the P/FeCo-NC catalyst displayed insignificant variation in current density for 10 h after holding at a constant potential of −0.04 V. The P/FeCo-NC catalyst showed little improvement in the HER overpotential by 9 mV at 10 mA cm^−2^ after 2000 cycles (Figure 6g). This result confirmed its excellent stability under acidic conditions. The chemical composition and structural evidence were studied through a durability test on P/FeCo-N-C catalyst. The test showed structural deviations with loss of N amount and the growth of metal agglomeration. This observation indicates a decrease in the active-site density and a decrease in activity.

Zhang et al. [65] synthesized SA Ni-N_4_ site-supported ruthenium nanoclusters (Ru NCs) for the enhancement in HER performance. A Ru/Ni-N_4_C-300 catalyst-based AEMWE device displayed higher performance than an electrolyzer loaded with 20% Pt/C. The AEMWE device (Ru/Ni-N_4_C-300 ‖ NiFe LDH) reached an industrial current density of 500 mA cm^−2^ with a low cell voltage of 1.72 V. Furthermore, the mass activity of the Ru/Ni-N_4_C-300 catalyst-based AEMWE device delivered ~2.46 and ~22.60 A/mg_Ru_ at 1.5 and 2.0 V, respectively. The AEMWE device (Ru/Ni-N_4_C-300 ‖ NiFe LDH) showed excellent durability for over 1370 h at 500 mA cm^−2^ under industrial conditions (1.0 M KOH at 50 °C) with insignificant degradation in performance.

Notably, post-reaction characterizations and post-operando studies are important for exploring the structural variations with catalytic performance and define more consistent structure–stability relationships for practical applications. Various degradation paths may contribute to loss of catalytic activity during long-term electrochemical operation, like migration and aggregation of isolated atoms and metal dissolution. These issues during operation alter the local coordination atmosphere and support-material degradation. Further, these conditions can modify the electronic structure, decrease the density of accessible active sites and reduce overall catalytic performance. Consequently, post-stability analysis investigations should be implemented to explore the dynamic evolution of active sites during long-term operation for their practical success.

Table 1 shows SAC-based HERs performed in previous studies with details on catalyst composition, mass activity, electrolyte, overpotential at 10 and 100 mA cm^−2^, reference electrode, mass loading, LSV operating condition, Tafel slope and durability.

**Figure 7:**
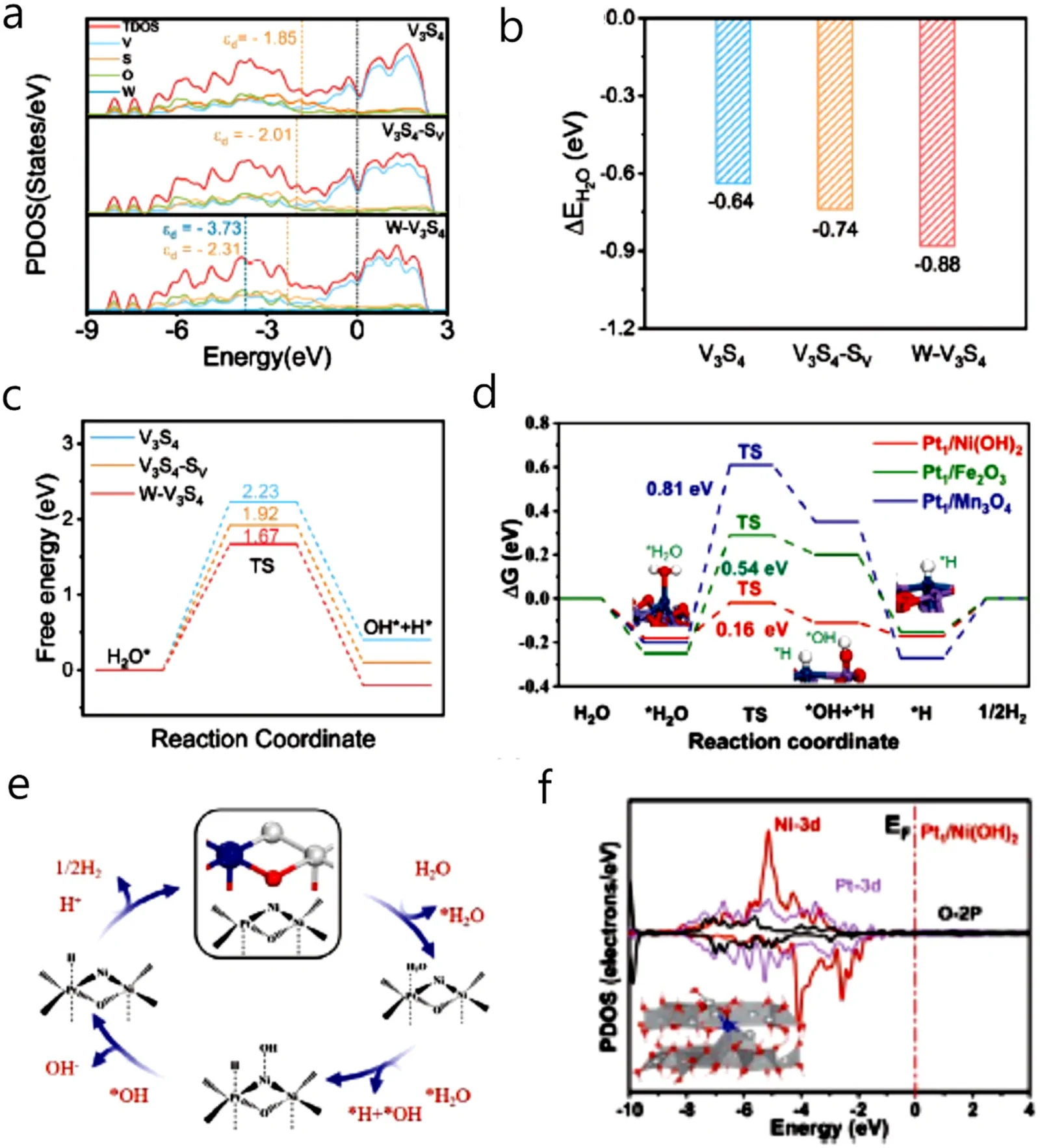
(**a**) DOS of the V_3_S_4_, V_3_S_4_-SV, and W-V_3_S_4_ systems; (**b**) estimated H_2_O adsorption energies of the V_3_S_4_, V_3_S_4_-SV, and W-V_3_S_4_ catalysts; (**c**) H_2_O dissociation paths of the V_3_S_4_, V_3_S_4_-SV, and W-V_3_S_4_ catalysts. Copyright 2025, Wiley-VCH Verlag GmbH & Co. KGaA, Weinheim [47]. (**d**) Energy barrier of H_2_O decomposition for Pt_1_/Ni(OH)_2_, Pt_1_/Fe_2_O_3_, and Pt_1_/Mn_3_O_4_ catalysts; (**e**) reaction path for the Pt_1_/Ni(OH)_2_ catalyst during the HER; (**f**) PDOS results for the Pt_1_/Ni(OH)_2_ catalyst. Copyright 2025, Wiley-VCH Verlag GmbH & Co. KGaA, Weinheim [81].

Xi’s group studied an S-vacancy defect-trapping approach to anchoring isolated W SAs onto ultrathin V_3_S_4_ nanosheets as effective nanocatalysts for the HER [47]. DFT calculations were performed to investigate the HER activity of the W-V_3_S_4_ catalyst. Figure 7a shows the projected density of states (PDOSs) of the V_3_S_4_, V_3_S_4_-SV, and W-V_3_S_4_ catalysts. The W-V_3_S_4_ catalyst delivered the highest electronic density of states (DOS) near the Fermi level, resulting in greater carrier density and enhanced intrinsic conductivity. For the W-V_3_S_4_ catalyst, the *d*-band centers of the W and V sites, which serve as active electronic descriptors, are −3.73 and −2.31 eV, respectively. These values are further from the Fermi level than those of the V sites in pristine V_3_S_4_ (−1.85 eV) and V_3_S_4_-SV (−2.01 eV), demonstrating substantial modulation of the electronic structure upon W SA incorporation, resulting in weaker adsorption of the reaction intermediates that create H_2_ on the surface of the catalyst. Consequently, the W-V_3_S_4_ catalyst showed enhanced HER activity because the incorporation of atomically dispersed W SAs successfully regulated the local electronic structure of V_3_S_4_ and improved the adsorption/desorption of hydrogen intermediates [82].

In addition, the synergistic balance between H_2_O adsorption and dissociation is an important process leading to the alkaline HER. The Δ*E*_H2O_ value of W-V_3_S_4_ (−0.88 eV) was lower (Figure 7b,c) than those of V_3_S_4_ (−0.64 eV) and V_3_S_4_-SV (−0.74 eV), representing stronger H_2_O adsorption on the W-V_3_S_4_ surface. Simultaneously, the energy barrier for H_2_O dissociation on W-V_3_S_4_ was ~1.67 eV, which was lower than those of V_3_S_4_ (~2.23 eV) and V_3_S_4_-SV (~1.92 eV), thereby allowing for effective H_2_O dissociation on the W-V_3_S_4_ surface. These findings confirm the noteworthy acceleration of the Heyrovsky step and establish the synergistic roles of the modulated active sites in the adsorption and dissociation of H_2_O molecules [83]. Theoretical calculations revealed that anchoring atomically dispersed W SAs efficiently induces charge density redistribution and improves the adsorption/desorption of reaction intermediates, thereby enhancing the HER catalytic performance [84].

An efficient HER catalyst comprising atomically dispersed Pt SAs confined within a Ni(OH)_2_ nanosheet lattice [(Pt_1_/Ni(OH)_2_)] was synthesized for high-performance HER by Cao et al. [81]. DFT calculations were performed to understand the cause of the altered activity of the Pt_1_/Ni(OH)_2_, Pt_1_/Fe_2_O_3_, and Pt_1_/Mn_3_O_4_ catalysts. Figure 7d,e show the energy barriers and reaction paths of the Pt_1_/Ni(OH)_2_, Pt_1_/Fe_2_O_3_, and Pt_1_/Mn_3_O_4_ catalysts. Pt_1_/Ni(OH)_2_ and Pt_1_/Mn_3_O_4_ exhibited weaker H_2_O adsorption energies, which facilitated the subsequent desorption of the dissociated OH* and H* intermediates. However, Pt_1_/Fe_2_O_3_ exhibited a higher H_2_O adsorption energy than Pt_1_/Ni(OH)_2_ and Pt_1_/Mn_3_O_4_ (Figure 7d). Therefore, the intermediate product was difficult to desorb, which is disadvantageous for continuous HER advancement. In this study, H_2_O dissociation at the Pt active sites was the rate-determining step. The total energy of H* at Pt sites (−583.27 eV) was lower than that of H* on the Ni sites (−583.02 eV). Therefore, H* was adsorbed more easily at the Pt active sites. In addition, Pt_1_/Mn_3_O_4_ required surmounting an energy barrier (~0.81 eV) to complete H_2_O dissociation (Figure 7d). The H_2_O dissociation energy barrier of Pt_1_/Fe_2_O_3_ (~0.54 eV) was less than that of Pt_1_/Ni(OH)_2_ (~0.16 eV). This result confirms that Pt_1_/Ni(OH)_2_ is more appropriate for the HER. In particular, Pt_1_/Ni(OH)_2_ delivered distinct active sites for hydroxyl (Ni sites) and hydrogen (Pt sites) adsorption, thereby facilitating H_2_O dissociation. The corresponding Δ*G*_H2O_ was reduced (Figure 7d) to ~0.16 eV on Pt_1_/Ni(OH)_2_, demonstrating its improved ability to cleave the HO-H [85]. Meanwhile, the Δ*G*_H*_ value of Pt_1_/Ni(OH)_2_ was −0.17 eV, which is very close to the ideal value (Δ*G*_H*_ = 0 eV). Therefore, the Pt atoms in the lattice-confined Pt_1_/Ni(OH)_2_ served as the active sites for the alkaline HER. The Pt atoms served as promoters of H_2_O dissociation, facilitating the generation of hydrogen intermediates. Moreover, the lattice-confined single Pt atoms on Ni(OH)_2_ further facilitated the subsequent H_2_ generation process. Figure 7e shows the reaction paths for the HER and Pt_1_/Ni(OH)_2_. DFT calculations revealed that the Pt 3*d* orbitals strongly hybridized with the O 2*p* and Ni 3*d* orbitals in Ni(OH)_2_, successfully regulating the electronic state of the Pt sites and significantly lowering the rate-determining step energy barrier for the HER to 0.16 eV. The electronic structures of the catalysts are shown in the PDOS (Figure 7f). Meanwhile, the strong orbital overlaps among the O-2*p*, Ni-3*d*, and Pt-3*d* orbitals serve as the electron source center for effective site-to-site electron transfer within the Pt_1_/Ni(OH)_2_, thereby enhancing its catalytic activity in the HER [86].

In addition, recent studies [87,88] demonstrate that electrolyte composition, solvation, applied potential and adsorbate-induced reconstruction can dynamically alter the coordination and electronic structure of single-atom sites, thereby changing their catalytic energetics and activity. Solvation effects may alter the adsorption strength and stabilization of key intermediates (like *H and *OH) through applied potential. Interfacial electric fields may change charge distribution and reaction energetics. Electrolyte composition and pH can affect water dissociation, the local reaction atmosphere and interfacial H^+^/OH^−^ transport. Consequently, adsorption energies and reaction barriers gained from idealized vacuum models should not be directly regarded as intrinsic descriptors under operating conditions. Current research [89] on dynamic reconstruction of Ni–N–C SACs highlights the potential-dependent evolution of single-atom coordination under electrochemical conditions. In future research, DFT-derived adsorption energies and reaction barriers should be carefully interpreted and completed by genuine interfacial models and operando characterization.

## 6. Synthesis Approaches for SACs

Scalable synthesis of SACs is essential to exploring effectiveness of catalysts from laboratory to practical applications [90,91]. Several synthesis approaches have been studied to enhance SAC content, like high-temperature pyrolysis treatment, ball-milling process and molten salt templating. These methods successfully delivered large-scale production of SACs via coordination environment monitoring, appropriate precursor plan, and optimization of the synthesis process, laying a solid material foundation for their practical employment. In addition, low-temperature synthesis and microwave heating provide unique synthesis procedures based on mild reaction environments and ultrafast heating. These synthesis approaches successfully suppress isolated single-metal-atom migration and agglomeration by allowing for the development of distinct low-coordination active sites. These facile and cost-effective synthesis approaches not only deliver paths for the scaling up of SAC amount but also create potential to increase metal content capacity, structural homogeneity and large-scale application research in SACs.

## 7. Conclusions and Future Perspectives

The transition toward sustainable and low-carbon energy systems requires developing effective technologies that can convert intermittent renewable energy into stable, storable chemical fuels. In this regard, H_2_ production via renewable-powered water electrolysis is imperative for achieving sustainable energy. The HER, as a cathodic half-reaction of water electrolysis, plays a key role in defining the overall energy efficiency of electrochemical H_2_ production. Therefore, the development of cost-effective, highly active/ultradurable, and scalable HER electrocatalysts is indispensable to converting laboratory-scale advances into practical H_2_ production technologies. The resulting hydrogen can be utilized in fuel cells, industrial processes, and other energy conversion technologies.

SACs offer excellent opportunities to maximize metal utilization by dispersing isolated metal atoms as catalytic centers. This atomic-level engineering enables enhancement of reaction pathways and the active tailoring of the adsorption/desorption energetics of key HER intermediates. In particular, the proper tuning of hydrogen adsorption free energy can enable the formation and successive release of H_2_. Water dissociation often represents an added kinetic barrier for the alkaline HER; SACs can encourage H_2_O adsorption and H-OH bond cleavage via judiciously designed metal centers, support interactions, and dual-site configurations. Strong coupling between atomically dispersed single atoms and their adjacent supports can further induce charge redistribution, orbital hybridization, and interfacial electronic interactions, enabling charge transfer and enhancing HER kinetics. In summary, SACs have advanced from simple isolated-metal configurations to sophisticated atomic architectures in which the metal center, coordination environment, support, interface, and local electronic structure together govern the catalytic performance.

Despite their substantial potential, numerous challenges remain before SACs can be applied in practical HER systems. First, isolated single metal atoms are thermodynamically inclined to aggregate, migrate, dissolve, or reconstruct themselves under electrochemical operating conditions. Maintaining SAC atomic dispersion during their long-term operation is challenging. Secondly, the catalytic properties of SACs are extremely sensitive to the local coordination atmosphere, metal–support interactions, and oxidation state, making precise synthesis and reproducible control difficult. Third, SACs under operational conditions may vary significantly from those characterized before electrolysis. Dynamic changes in coordination number, adsorption configuration, oxidation state, and adjacent atoms create active sites during the HER. Fourth, the very low metal loading of SACs restricts the total number of active sites. Many studies on SACs have focused on comparatively low current densities and short-term laboratory-scale measurements, whereas industrial electrolyzers require long-term operation, high current densities, low catalyst loadings, and stable electrode/electrolyte interfaces. Finally, the high cost and restricted abundance of SACs remain important considerations for large-scale deployment.

Further, important scientific challenges and practical limitations persist unanswered, needing serious assessment to direct future research toward efficient and feasible hydrogen production. (i) Active-site identification and evolution: The accurate oxidation state and coordination environment of isolated single metal atoms during operation remain inadequately understood. Therefore, operando confirmation should be explored to know the initial structures or dynamic reconstruction of the active sites in operating environments. (ii) Activity-stability trade-offs: Metal–support interactions (MSIs) can suppress single-atom aggregation and migration. In addition, MSIs may change energetics of hydrogen adsorption and decrease catalytic performance. Systematic approaches remain a crucial challenge to attain excellent activity and stability. (iii) Structure–activity relationships: The specific contributions of coordination number, adjacent atoms’ metal individuality and metal–support interactions to hydrogen adsorption and HER kinetics remain difficult to differentiate experimentally. (iv) Scalability and cost: Reproducible synthesis is another major challenge for preparing homogeneously dispersed isolated single atoms in the same amount. Furthermore, reducing precious-metal content remains a key challenge for controlling the cost of SACs for the HER. (v) Consistent performance evaluation: Variations in catalyst amount, electrode architecture, accessible surface area and testing conditions complicate comparisons across electrochemical studies. Three-electrode outcomes alone cannot provide real electrolyzer performance.

The potential of SACs in membrane–electrode assemblies (MEAs) is indispensable to developing the practical significance of the SAC-based HER. This review describes the effectiveness of SACs in MEAs in Figure 6. Further, research investigations should explore current density, cell voltage, SAC content and prolonged durability under realistic operating circumstances by monitoring mass-transport restrictions, interfacial barriers, and compatibility of catalyst layer and membrane. In addition, SACs for the HER should be assessed at industrially applicable current densities (typically ≥500 mA cm^−2^) for practical H_2_ production rather than laboratory benchmarks. Further, the long-term stability of the catalysts needs to explore the degradation mechanisms of SACs, including atom aggregation, migration, support corrosion, leaching and changes in coordination surroundings during prolonged operation. These issues can be addressed by engineering well-distributed anchoring sites and metal–support interactions, enhancing metal content and retaining corrosion-resistant single-atom supports. Confinement within appropriate porous structures further control atomic aggregation/migration, and stable coordination surroundings can decrease metal dissolution under long operation. Thus, engineering structural morphology and their electronic coordination are described in Section 3 and Section 4. Several research methods, like wet-chemical impregnation, controlled pyrolysis and continuous-flow processing are possible ways to increase the quantity of single metals in the catalysts and to reduce the overall cost of catalyst design. In addition, the formation of defects and vacancies in the catalysts prevents migration during long-term operation. Besides, facile synthesis procedures, effective precursor control, and cost-effective support materials are indispensable to economic feasibility. Developing scalable, reproducible synthesis methods with high atomic utilization, minimal processing steps, and reduced precious-metal consumption is essential to lowering manufacturing costs and facilitating commercial deployment.

Therefore, future SAC development should move beyond simply increasing their intrinsic activity toward the simultaneous optimization of their activity, stability, atomic utilization, and scalability. Lattice confinement, defect engineering, heteroatom coordination, and spatially controlled anchoring can provide stable environments for isolated-metal centers. Simultaneously, self-supported electrodes can increase active-site accessibility while facilitating electrolyte penetration, gas transport, and electron conduction. Development of dual-atom and multi-atomic configurations may provide an additional strategy to overcome the limitations of isolated single sites by creating cooperative reaction centers while maintaining atomic-level structural control. Similarly, integrating SACs with conductive supports, heterostructures, or multifunctional interfaces can simultaneously regulate the electronic structure and improve mass/electron transport.

Future SAC-based HER research should drive towards monitoring active-site closeness, dynamic reconstruction and local coordination from just maximizing isolated single-atom distribution. Current research challenges the conventional statement that maximum spatial isolation of single atoms is always best for the HER. Chen et al. [92] established that adjacent single-Pt sites showed 41-fold higher mass activity compared to isolated Pt sites, which was ascribed to the development of a Pt-H-Pt bridge intermediate that enables H–H coupling. Zhai’s research group [93] reported that isolated SACs contained up to 7.85 wt% of single-Pt-atom sites. In addition, catalysts delivered ~3 A cm^−2^ at 1.804 V in a PEM water electrolyzer at 80 °C. These findings represent excellent single-atom density and practical electrode architectures. Therefore, future SAC design should improve site density, interatomic distance and coordination rather than just atomic dispersion. At the same time, electronic structure under prolonged operation and active sites that dynamically change coordination are equally vital to high-performance SACs [94]. Subsequently, operando characterization along with DFT/ML-guided modeling should explore the accurate working state of active sites for SACs. Thus, SAC-based HER performance progress should focus on high metal loading, industrial current density, prolonged durability, and electrolyzer-level corroboration over conventional three-electrode systems.

The coalescence of isolated single metal atoms can be efficiently controlled by monitoring the nature and density of anchoring sites like defects, heteroatom coordination surroundings and vacancies, which deliver significant metal–support interactions (MSIs) and reduce their surface mobility. In addition, the strong coordination among the atomically dispersed single-metal centers and adjacent heteroatoms improves their thermodynamic stability and decreases coalescence throughout electrochemical operation. Therefore, optimizing the spatial distribution of vacancies, along with the synthesis conditions and concentration of the metal precursors, is important for accomplishing stable and highly dispersed single-atom sites to keep atomically dispersed active metal centers under long-term reaction conditions.

A key future direction is to integrate operando/in situ characterization, DFT analysis, and machine learning (ML) to obtain a comprehensive understanding of SACs under realistic HER conditions. Operando X-ray absorption spectroscopy, Raman spectroscopy, infrared spectroscopy, photoelectron spectroscopy, and electron microscopy can provide valuable information regarding changes in the oxidation state, coordination environment, adsorption configuration, and structural reconstruction during catalysis. Such experimental observations are indispensable to recognizing real active sites rather than relying exclusively on ex situ structural characterization. Future computational studies should gradually incorporate realistic electrolyte atmospheres, applied potentials, solvation effects, and dynamic structural evolutions to narrow the gap between theoretical models and experimental systems. ML offers promising opportunities by linking large datasets of SAC compositions, electronic descriptors, coordination structures, adsorption energies, and catalytic performance. ML-assisted screening can accelerate the identification of promising metal–support combinations and disclose nonlinear associations that can create a closed-loop approach for high-performance SAC discoveries.

Overall, the future development of SACs for the HER should progress from the conventional concept of “isolated metal atoms as active sites” toward a more comprehensive understanding of dynamic atomic catalytic systems. Future SACs should be rationally designed by combining advanced synthesis with predictive DFT calculations, data-driven ML, and operando characterizations that could allow the finding of SACs with precisely measured catalytic properties. Finally, SAC-based HER electrocatalysis has the potential to contribute significantly to renewable electricity-to-hydrogen conversion, long-term chemical energy storage, and low-carbon energy conversion technologies.

## Figures and Tables

**Table 1:** SAC-based HER under standard three-electrode systems: catalyst composition, mass activity, electrolyte, overpotential at 10 and 100 mA cm^−2^, reference electrode, mass loading, LSV operating condition, Tafel slope and durability.

Catalyst Composition	Reference Electrode/Mass Loading	Mass Activity	Electrolyte and Operating Condition for LSV	Overpotential at 10 and 100 mA cm^−2^	Tafel Slope	Durability	Ref.
Pt SAC-NiCrO_3_/NF	Hg/HgO	Mass activity: ~382.77 mA mg^−1^_Pt_ (overpotential is 100 mV; capacity of Pt is 0.21 mg cm^−2^)	1 M KOH and LSV polarization curves studied at scan rate (5 mV s^−1^), incorporating 85% iR compensation	23 and 122 mV	38.73 mV dec^−1^	Performance decay is negligible after 1000 cycles operating at 100 mA cm^−2^	[43]
P/FeCo-NC	Hg/Hg_2_SO_4_ Catalyst loading of ~0.25 mg cm^−2^.	----	0.5 M H_2_SO_4_; LSV was done at 2 mV s^−1^ scan rate in Ar-saturated electrolyte solution with 95% iR-correction	38 mV at 10 mA.cm^−2^	37 mV dec^−1^	Time-dependent current density curve displayed insignificant change after holding at constant potential of −0.04 V for 10 h at 10 mA cm^−2^	[51]
Ru_SA_-NiCoP 3D porous, flower-like catalysts	Hg/HgO	---	1 M KOH and LSV polarization curves at a scan rate of 5 mV s^−1^, incorporating 85% iR compensation	16 and 99 mV	41.2 mV dec^−1^	Robust stability over 80 h at 100 mA cm^−2^	[55]
Ru/Ni-N_4_C-300	Hg/HgO Loading of catalyst was ~1 mg cm^−2^	Mass activity at 100 mV is 3.2 A mg^−1^ (normalized by the amount of Ru)	1 M KOH; LSV polarization plot at 5 mV s^−1^ scan rate, incorporating 95% iR compensation	15 mV at 10 mA cm^−2^	23.7 mV dec^−1^	Negligible performance degradation after constant operation over 20 h at 10 mA cm^−2^	[65]
W_1_O/NiS	Hg/HgO	----	1 M KOH and LSV polarization study done at 5 mV s^−1^ scan rate.	76 mV at 10 mA.cm^−2^	77 mV dec^−1^	98% stability after 300 h at 200 mA cm^−2^	[79]
Lattice-confined Pt_1_/Ni(OH)_2_	Calomel	Mass activity of 6746 mA/mg_Pt_ under overpotential of 500 mV (normalized by the mass loading of Pt)	1 M KOH; LSV polarization plot studied at 2 mV s^−1^ scan rate	15 mV at 10 mA.cm^−2^	65 mV dec^−1^	No clear degradation after 5000 cycles. It showed stable durability under current density of 800 mA cm^−2^, and the potential needed at 800 mA cm^−2^ changes slightly after 400 h.	[81]

## Data Availability

No new data were created or analyzed in this study. This article is based on a comprehensive review of the previously published literature. All data supporting the findings of this study are available within the article and in the cited references. And copyright permission was taken from the corresponding journals.

## Abbreviations

The following abbreviations are used in this manuscript:

AC	aberration-corrected
AEMWE	anion exchange membrane water electrolyzer
AWE	anion water electrolyzer
CV	cyclic voltammetry
DFT	density functional theory
DOS	density of states
EDS	energy-dispersive spectroscopy
EIS	electrochemical impedance spectroscopy
EXAFS	extended X-ray absorption fine structure
FE	faraday efficiency
HAADF	high-angle annular dark-field
HER	hydrogen evolution reaction
IR	infrared
LSV	linear sweep voltammetry
LVS	lattice vacancy site
ML	machine learning
NS	nanosheet
OER	oxygen evolution reaction
PDOS	projected density of states
PEM	proton exchange membrane
SA	single atom
SAC	single-atom catalyst
SRPES	synchrotron radiation photoemission spectroscopy
XANES	X-ray absorption near-edge structure
XPS	X-ray photoelectron spectroscopy
XRD	X-ray diffraction
PNF	PtNiFe (catalyst)
STEM	scanning transmission electron microscopy
TEM	transmission electron microscopy
